# Propagation path of a flowering cherry (*Cerasus* × *yedoensis*) cultivar ‘Somei-Yoshino’ traced by somatic mutations

**DOI:** 10.1093/dnares/dsae025

**Published:** 2024-08-08

**Authors:** Kenta Shirasawa, Tomoya Esumi, Akihiro Itai, Katsunori Hatakeyama, Tadashi Takashina, Takuji Yakuwa, Katsuhiko Sumitomo, Takeshi Kurokura, Eigo Fukai, Keiichi Sato, Takehiko Shimada, Katsuhiro Shiratake, Munetaka Hosokawa, Yuki Monden, Makoto Kusaba, Hidetoshi Ikegami, Sachiko Isobe

**Affiliations:** Department of Frontier Research and Development, Kazusa DNA Research Institute, Kisarazu, Japan; Academic Assembly Institute of Agricultural and Life Sciences, Shimane University, Matsue, Japan; Department of Agricultural and Life Science, Kyoto Prefectural University, Kyoto, Japan; Faculty of Agriculture, Iwate University, Morioka, Japan; Horticultural Research Institute, Yamagata Integrated Agricultural Research Center, Sagae, Japan; Yamagata Nishi High School, Yamagata, Japan; Institute of Vegetable and Floriculture Science, NARO, Tsukuba, Japan; Faculty of Agriculture, Utsunomiya University, Utsunomiya, Japan; Graduate School of Science and Technology, Niigata University, Niigata, Japan; Yamanashi Kofu Minami High School, Kofu, Japan; Institute of Fruit Tree and Tea Science, NARO, Shizuoka, Japan; Graduate School of Bioagricultural Sciences, Nagoya University, Nagoya, Japan; Department of Agricultural Science, Kindai University, Nara, Japan; Graduate School of Environmental and Life Science, Okayama University, Okayama, Japan; Graduate School of Integrated Sciences for Life, Hiroshima University, Higashi-Hiroshima, Japan; Resident, Fukuoka, Japan; Department of Frontier Research and Development, Kazusa DNA Research Institute, Kisarazu, Japan

**Keywords:** clone, flowering cherry, genome sequence, somatic mutation, Somei-Yoshino

## Abstract

In the long history of human relations with flowering cherry trees in Japan, ‘Somei-Yoshino’ occupies an exceptional position among a variety of flowering trees: it is a self-incompatible interspecific hybrid but has been enthusiastically planted by grafting throughout Japan, due most likely to its flamboyant appearance upon full bloom. Thus, ‘Somei-Yoshino’ gives us a rare opportunity to trace and investigate the occurrence and distribution of somatic mutations within a single plant species through analysis of the genomes of the clonally propagated trees grown under a variety of geographical and artificial environments. In the studies presented here, a total of 46 samples of ‘Somei-Yoshino’ trees were collected and their genomes were analysed. We identified 684 single nucleotide mutations, of which 71 were present in more than two samples. Clustering analysis of the mutations indicated that the 46 samples were classified into eight groups, four of which included 36 of the 46 samples analysed. Interestingly, all the four tree samples collected in Ueno Park of Tokyo were members of the four groups mentioned above. Based on comparative analysis of their mutations, one of the four trees growing in Ueno Park was concluded to be the closest to the original ancestor. We propose that somatic mutations may be used as tracers to establish the ancestral relationship amongst clonally propagated individuals.

## Introduction

More than 200 natural and domesticated cultivars of flowering cherry trees are said to exist in Japan. Of them, the most popular cultivar is named ‘Somei-Yoshino’, which is a self-incompatible interspecific hybrid between *Cerasus spachiana* and *C. speciosa*^1,2^ produced some 200 years ago.^3^ In the long history of human relations with these flowering cherry trees in Japan, ‘Somei-Yoshino’ is known to have frequently been chosen to be planted in parks, gardens, river banks, and various other places, largely because of its flowering characteristics: it may often be observed that almost an entire tree of ‘Somei-Yoshino’ is covered by thousands of tiny white flowers at the time of full bloom as if they inform visitors of the arrival of spring. The south to north migrating zone of blooming ‘Somei-Yoshino’ trees along the Japanese archipelago is often referred to as ‘the flowering cherry front’ which is daily forecasted during the season.^4,5^ Thus, ‘Somei-Yoshino’ is a best-known example of flowering trees favoured by people for planting almost everywhere throughout Japan.

‘Somei-Yoshino’ possesses a highly heterozygous genome, partly because it is an interspecific hybrid.^1,2^ As it has self-incompatibility as well as the high-heterozygous genome due to the interspecific hybrid, ‘Somei-Yoshino’ has been propagated mainly by grafting.^6^ Whether ‘Somei-Yoshino’ emerged naturally or it was artificially created remains unknown except that its origin was somehow related to a village named Somei in Tokyo and its existence was known at the end of the Edo period some 150 years ago.^3^ Studies suggest that the original tree of ‘Somei-Yoshino’ was planted either in Koishikawa Botanical Garden (Tokyo, Japan),^7^ Ueno Park (Tokyo, Japan)^8^, or Kaiseizan Park (Fukushima, Japan).^9^ Despite the note of Nakamura et al.^8^ who, based on the sequence analysis of *PolA1* gene, suggested that there are sufficient genetic resources to create ‘Somei-Yoshino’ in Ueno Park, details are still unknown. The origin of ‘Somei-Yoshino’ thus remains an interesting issue for researchers as well as people who are fascinated with ‘Somei-Yoshino’ and other cherry species.

Somatic mutations sometimes occur in different organs of an organism and are rarely eliminated, leading to chimerism.^10–12^ Some of the mutations in genes affect their functions, resulting in phenotypic alterations.^13^ Bud sports may be found in branches of trees as a result of somatic mutations.^13,14^ Since bud sports are genetically stable, grafting or cutting is used to propagate the mutants into new cultivars.^14^ Furthermore, silent somatic mutations, which do not affect gene functions nor cause phenotypic variations, also exist and are stably inherited. Therefore, somatic mutations may be used to trace the history of a clonally propagated plants such as ‘Somei-Yoshino’.

Considering these, we thought that ‘Somei-Yoshino’ would give us a rare opportunity to trace and investigate the occurrence and distribution of somatic mutations through analysis of the genomes of the clonally propagated ‘Somei-Yoshino’ trees planted in different environments under a variety of geographical and artificial conditions such as heavy snow- and/or rain-fall, different ambient temperatures and humidities, interactions with other plants, birds, animals, insects etc. The clonality of ‘Somei-Yoshino’ has been investigated by DNA fingerprinting using microsatellite markers.^1,6^ In addition, somatic mutations within a single ‘Somei-Yoshino’ tree were studied using techniques such as temperature gradient gel electrophoresis (TGGE)^15^ and double-digest restriction site-associated DNA sequencing (ddRAD-Seq).^16^ These studies indicated that the genetic identity of ‘Somei-Yoshino’ is high, although quite a few somatic mutations were detected. The genome (2*n* = 16) of ‘Somei-Yoshino’ (Tree ID #136 in Ueno Park) has been sequenced at the haplotype-phased chromosome level and 95,076 genes were identified in the 16 chromosome sequences spanning 690.1 Mb in total.^2^ Here, we report the whole-genome sequence analysis of somatic mutations in the 46 ‘Somei-Yoshino’ tree samples to characterize and establish their mutual relations.

## Materials and methods

### Plant materials

Leaves were collected from 46 ‘Somei-Yoshino’ trees grown in 19 prefectures of Japan (Supplementary Table S1); these 46 trees included the four trees, tree IDs of #133, #134, #136, and #138 planted in Ueno Park, Tokyo, Japan, which geographical positions are available in Nakamura et al.^8^ Genomic DNA was extracted from the leaves using the FavorPrep Plant Genomic DNA Extraction Mini Kit (Favorgen, Ping-Tung, Taiwan).

### DNA sequencing

Genomic DNA libraries were prepared with a PCR-free method using the Swift 2S Turbo Flexible DNA Library Kit (Swift Biosciences, Ann Arbor, MI, USA), and converted into a DNA nanoball sequencing library with the MGI Easy Universal Library Conversion Kit (MGI Tech, Shenzhen, China). The library was sequenced on the DNBSEQ G400RS (MGI Tech) instrument in paired-end, 150 bp mode. The sequence data of tree #136, with accession numbers DRR169775 (Sample name of SyTKY0 in this study) and DRR169776 (SyTKY1), were obtained from a DNA database.

### Detection and analysis of somatic mutations

Low-quality bases (quality score < 10) and adaptor sequences (AGATCGGAAGAGC) were trimmed with PRINSEQ (version 0.20.4)^17^ and fastx_clipper, respectively, in FASTX-Toolkit (version 0.0.14) (https://github.com/agordon/fastx_toolkit), and the remaining high-quality reads were mapped on to the haplotype-resolved chromosome-level genome sequence of ‘Somei-Yoshino’ (CYE_r3.1.pseudomolecule, obtained from DBcherry: https://cherry.kazusa.or.jp)^2^ with Bowtie 2 (version 2.3.5.1).^18^ The resultant SAM files were converted into BAM files with the *view* command of SAMtools (version 0.1.19).^19^ The gVCF files were generated from the BAM files using the *mpileup* command (-Ou -a DP,AD,INFO/AD) and *call* command (-Oz -m -g 0,10) in BCFtools (version 1.9),^19^ and normalized using the *norm* command of BCFtools. The individual gVCF files were merged into a single VCF file using the *merge* command of BCFtools. High-confidence, biallelic, homozygous single nucleotide variants (SNVs) were selected with VCFtools (version 0.1.12b).^20^ Genome positions of the SNVs were visualized with MapChart (version 2.2).^21^ Copy number variations (CNVs) were detected with cnv-seq (version 0.2.7),^22^ and the effect of sequence variations on gene function was predicted with SNPeff (version 4.2).^23^

### Cultivar identification

To identify cultivars of the tested 46 samples, the ddRAD-Seq data of 139 lines were downloaded from a DNA database (GenBank accession numbers: DRR169804–DRR169942). High-quality reads, selected as described above, were mapped on to the genome sequence of ‘Somei-Yoshino’ (CYE_r3.1.pseudomolecule), and SNVs were detected as described previously.^2^ SNVs identified in the 46 samples, based on the analysis of ddRAD-Seq reads and gVCF files, were combined to be subject to a principal component analysis (PCA). A phylogenetic tree based on 100 bootstrap replicates was created with SNPhylo (version 20140701),^24^ in which *Padus grayana* Uwamizu-zakura (Cerasus_2-58) was employed as an outgroup, and visualized with iTOL (version 6.9.1).^25^ To estimate the optimal number of clusters, the cumulative explained variance was calculated with Tassel 5.^26^

### SNV analysis of ancestral *Cerasus*

To identify the possible ancestral alleles of SNVs, whole-genome sequence reads were obtained from a public DNA database (GenBank accession numbers: DRR169795–DRR169803 and SRR6957274)^2^ for 10 lines: *C. campanulata* (Kanhi-zakura [Cerasus_1-57]); *C. serrulata* (‘Azumanishiki’ [Cerasus_2-51], ‘Gyoikou’ [Cerasus_2-19], ‘Ichihara-toranowo’ [Cerasus_2-27], and ‘Senrikou’ [Cerasus_1-37]); *C. spachiana* (‘Yaebeni-shidare’ [Cerasus_1-43]); *C. speciosa* (Ohshima-zakura [Cerasus_1-71]); *C. jamasakura* (Yama-zakura); *C.* × *nudiflora* (‘Eishu-zakura’); and *Cerasus* sp. (‘Oshidori-fujizakura’ [Cerasus_2-25]). SNV detection was performed as above. Major alleles across the 10 lines were presumed to be ancestor-type alleles, and the ancestor-type alleles in each of the 46 ‘Somei-Yoshino’ samples were counted.

## Results

### Cultivar identification

**Figure 1.**
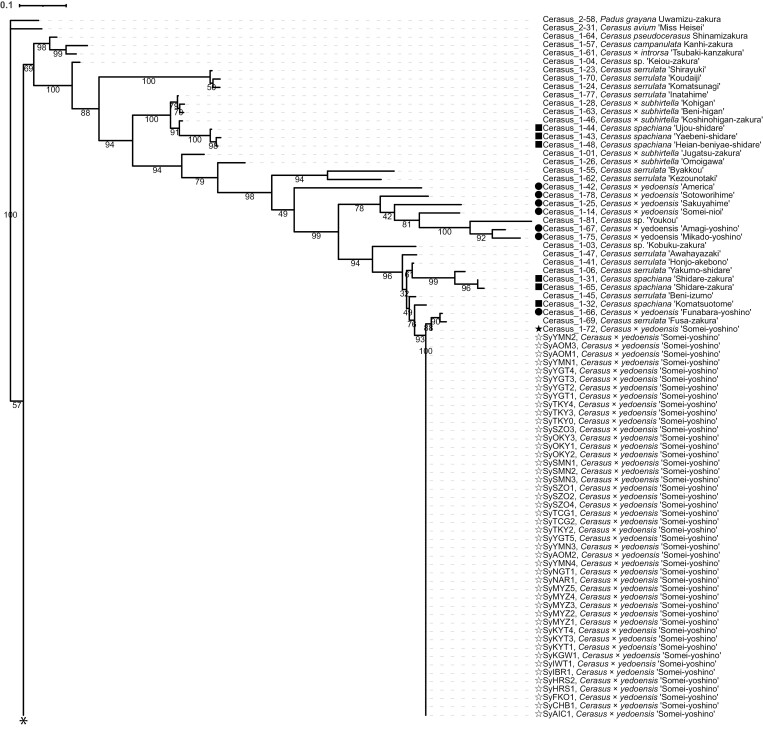

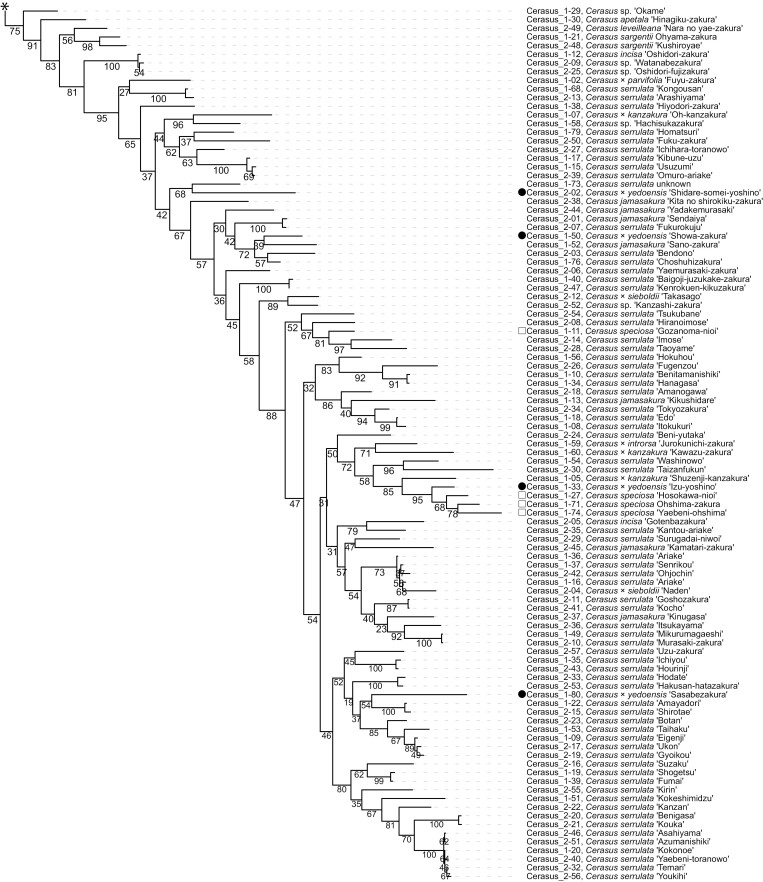
Phylogenetic tree of flowering cherry lines. Phylogenetic tree of 46 ‘Somei-Yoshino’ clones from this study and 139 cherry lines from our previous study.^2^ Black and white stars indicate the ‘Somei-Yoshino’ tree (Cerasus_72-1) used in our previous study^2^ and 46 clones used in this study, respectively. Black circles show synthetic hybrids, *Cerasus* × *yedoensis*, generated by crossing *C. spachiana* and *C. speciosa*.^3^ Black and white squares indicate probable parental lines of *C. spachiana* and *C. speciosa*, respectively. Unlabeled lines are non-relatives of ‘Somei-Yoshino’. Numbers on branches indicate bootstrap values based on 100 replicates. Asterisk indicates the connecting point of the branch.

To confirm the 46 samples used in this study were indeed ‘Somei-Yoshino’ clones, a clustering analysis based on genotypes of the 46 ‘Somei-Yoshino’ samples and 139 *Cerasus* lines. Whole-genome sequence reads obtained from 46 samples (mean depth of 34× genome coverage: Supplementary Table S1) and ddRAD-Seq reads obtained from 139 lines in our previous study,^2^ including ‘Somei-Yoshino’ (Sample ID of Cerasus_1-72 in the previous study), were aligned on to the reference genome sequence of ‘Somei-Yoshino’. A total of 5,328 SNPs were detected across the 185 samples, and genetic distances between each pair of the 185 samples were calculated to create a dendrogram (Fig. 1, Supplementary Table S2). As expected, the 46 samples and ‘Somei-Yoshino’ (Cerasus_1-72) clustered together, while the other lines including probable sister (*Cerasus* × *yedoensis*) and parent (*C. spachiana* and *C. speciosa*) lines were distinguishable from ‘Somei-Yoshino’. The result indicated that the 46 samples used in this study were genetically identified as ‘Somei-Yoshino’ clones.

### Detection and characterization of somatic mutations

**Figure 2.**
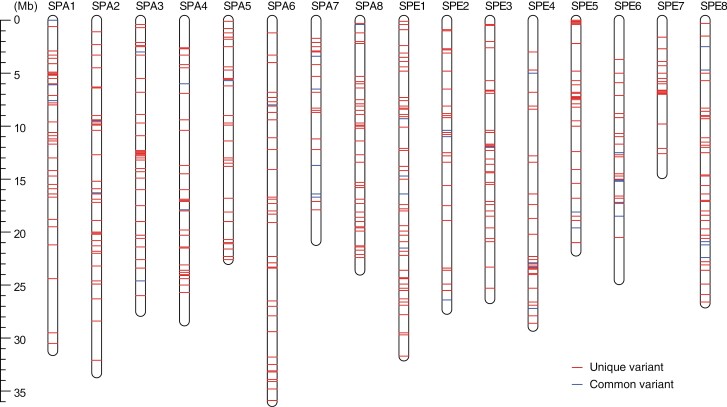
Genomic position of the 684 somatic mutations. SPA1–SPA8 and SPE1–SPE8 indicate the chromosomes of ‘Somei-Yoshino’.

**Figure 3.**
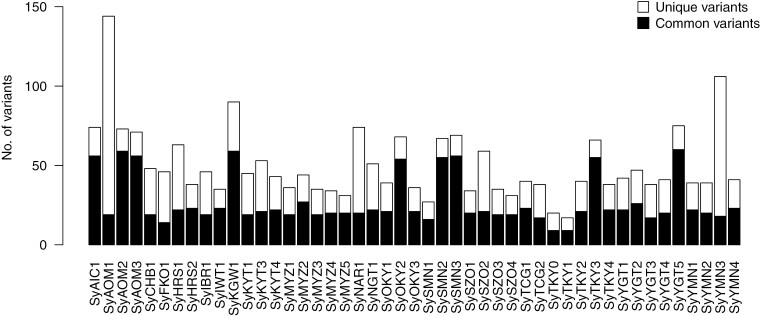
Numbers of variants in the 46 ‘Somei-Yoshino’ clones. Black and white bars show numbers of common and unique variants, respectively.

Based on the whole-genome sequence analysis of the 46 ‘Somei-Yoshino’ samples, 80,334 sequence variant candidates were detected. First, we selected 35,757 biallelic SNVs since tri- and tetra-allelic SNVs are generally rare and probably due to mapping errors in and near repeated sequences.^27^ Next, 1,942 sites were selected, on which only reads from haplotype-specific alleles were mapped on the haplotype-resolved phased genome sequence of ‘Somei-Yoshino’. Then, 1,749 single nucleotide variants, whose genotypes were consistent among the biological replicates of SyTKY0 and SyTKY1, were retained. Finally, we applied two filtering criteria, namely, read depth (≤ 50) and quality (≥ 80), to select 684 high-confidence SNVs, which were evenly distributed across the genome (Fig. 2, Supplementary Table S3). The number of variants across the 46 samples was 50.3, on average, with the maximum value of 144 in SyAOM1, followed by 106 in SyYMN3 and 90 in SyKGW1 (Fig. 3, Supplementary Tables S1 and S3). The 684 SNVs consisted of 285 C/G to T/A transitions (41.7%), 122 A/T to T/A transversions (17.8%), 120 A/T to G/C transitions (17.5%), 72 C/G to A/T transversions (10.5%), 51 C/G to G/C transversions (7.5%), and 34 A/T to C/G transversions (5.0%). The transition/transversion ratio was 1.45. Among the 684 variants, 88 variants (12.9%) were in gene bodies, whereas the remaining 596 variants (87.1%) were found in intergenic regions.

Of the 684 variants, 613 were unique to a single clone and 71 were common to at least two clones (Fig. 3, Supplementary Tables S1 and S3). Of the 88 variants identified in gene bodies, seven variants, all of which were unique to a single clone, were predicted to have a high impact on gene functions (Table 1, Supplementary Tables S3 and S4). These seven variants included four nonsense mutations (in CYE_r3.1SPA5_g009120 in SyYMN2, CYE_r3.1SPE0_g067760 in SyYGT3, CYE_r3.1SPE5_g007060 in SyIBR1, and CYE_r3.1SPE8_g002010 in SyYMN3), two mutations at splice acceptor sites (in CYE_r3.1SPA4_g020770 in SyNGT1 and CYE_r3.1SPA5_g023100 in SyYGT4), and one at a splice donor site (in CYE_r3.1SPE0_g037740 in SyYMN3). Missense mutations were found in 23 genes (Supplementary Tables S3 and S4).

**Table 1.** Somatic mutations highly affecting gene functions

Gene ID	Mutation	Description
CYE_r3.1SPA4_g020770	Splice acceptor variant and intron variant	[Y4729_ARATH] G-type lectin S-receptor-like serine/threonine protein kinase At4g27290
CYE_r3.1SPA5_g009120	Stop gained	[ARC_HUMAN] Activity-regulated cytoskeleton-associated protein
CYE_r3.1SPA5_g023100	Splice acceptor variant and intron variant	[SIS3_ARATH] E3 ubiquitin-protein ligase SIS3
CYE_r3.1SPE0_g037740	Splice donor variant and intron variant	[RNHX1_ARATH] Putative ribonuclease H protein At1g65750
CYE_r3.1SPE0_g067760	Stop gained	[POLX_TOBAC] Retrovirus-related Pol polyprotein from transposon TNT 1-94
CYE_r3.1SPE2_g031860	Gene deletion	[LBD29_ARATH] LOB domain-containing protein 29
CYE_r3.1SPE2_g031870	Gene deletion	[GRD2I_MOUSE] Delphilin
CYE_r3.1SPE2_g031880	Gene deletion	No hits
CYE_r3.1SPE5_g007060	Stop gained	[ELH1_APLCA] ELH
CYE_r3.1SPE6_g015220	Gene deletion	[DRL36_ARATH] Probable disease resistance protein At5g45510
CYE_r3.1SPE6_g015230	Gene deletion	[CITG2_SALTY] Probable 2-(5ʹ-triphosphoribosyl)-3ʹ-dephosphocoenzyme-A synthase 2
CYE_r3.1SPE8_g002010	Stop gained	[LKHA4_DEBHA] Leukotriene A-4 hydrolase homolog
CYE_r3.1SPE8_g017860	Gene deletion	[AB2A_ARATH] ABC transporter A family member 2

### CNVs among the ‘Somei-Yoshino’ clones

**Figure 4.**
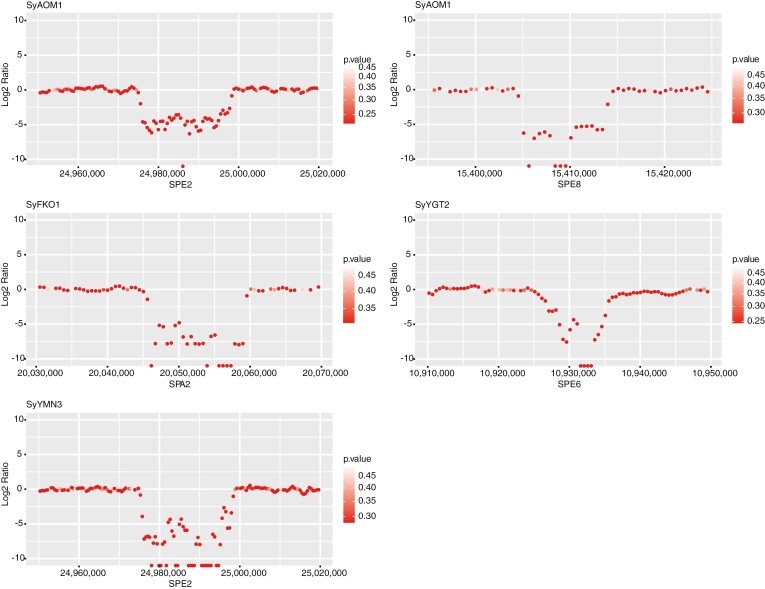
Copy number variations (CNVs) detected in the four ‘Somei-Yoshino’ clones. Clone and chromosome names are shown above and below the plots, respectively. Numbers on x-axes indicate chromosome positions (bp). The log2 ratio on y-axes above zero indicates insertions in the tested lines with respect to that of TKY0 as a reference line. The *P*-values are calculated as a probability of a copy number ratio being divergent from 1:1 ratio by a random chance. Details are mentioned by Xie and Tammi (2009).

At least five CNVs were found in four clones, SyAOM1, SyFKO1, SyYGT2, and SyYMN3 (Fig. 4, Table 1, Supplementary Table S4). All five CNVs were deletion mutations with respect to SyTKY1 as a standard; SyAOM1 carried two CNVs, one on chromosome SPE2 (~25 kb deletion) and another on chromosome SPE8 (~10 kb deletion), while each of the three remaining clones, SyFKO1, SyYGT2, and SyYMN3, carried a single deletion on chromosomes SPA2 (~15 kb deletion), SPE6 (~10 kb deletion), and SPE2 (~25 kb deletion), respectively. Among these CNVs, the deletions on chromosome SPE2 in SyAOM1 and SyYMN3 might be identical. The deletions encompassed three genes on chromosome SPE2 (CYE_r3.1SPE2_g031860, CYE_r3.1SPE2_g031870, and CYE_r3.1SPE2_g031880), one gene on SPE8 (CYE_r3.1SPE8_g017860), and two genes on SPE6 (CYE_r3.1SPE6_g015220 and CYE_r3.1SPE6_g015230), while the deletion on SPA2 contained no gene.

### Clustering analysis of ‘Somei-Yoshino’ clones

**Figure 5.**
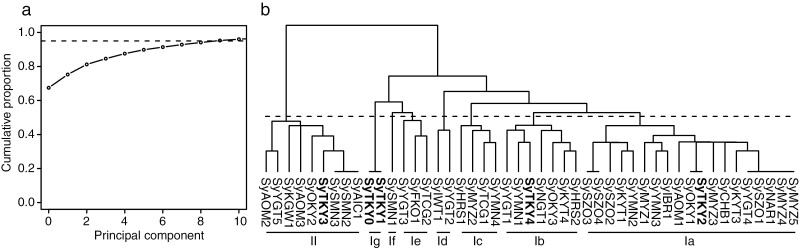
Clusters of 46 ‘Somei-Yoshino’ clones. (a) Cumulative explained variance in the principal component analysis. A horizontal dotted line indicates 0.95 of cumulative explained variance. (b) Dendrogram of 46 ‘Somei-Yoshino’ clones. Boldface indicates four trees planted in Ueno Park (Tokyo, Japan). SyTKY0 and SyTKY1 represent different sequence datasets obtained from the same tree (#136). Group names (Ia–Ie and II) are shown below the dendrogram.

The cumulative explained variance was calculated with the 71 common variants (Fig. 5a). When the threshold value was set to 0.95, a cluster number of 8 was the smallest value of the cluster with a cumulative explained variance ratio. Therefore, the 46 samples were divided into eight groups, including two major groups (I and II) with seven subgroups (Ia to Ig) (Fig. 5b). The numbers of clones in each group were as follows: 19 (Ia), 7 (Ib), 4 (Ic), 2 (Id), 3 (Ie), 1 (If), 1 (Ig), and 9 (II). These clusters showed no correlation with the sample collection site. Each of the four clones collected from Ueno Park (Tokyo, Japan), SyTKY1–SyTKY4, grouped into four different clusters (Ia, Ib, Ie, and II), which contained 38 of the 46 clones tested.

**Figure 6.**
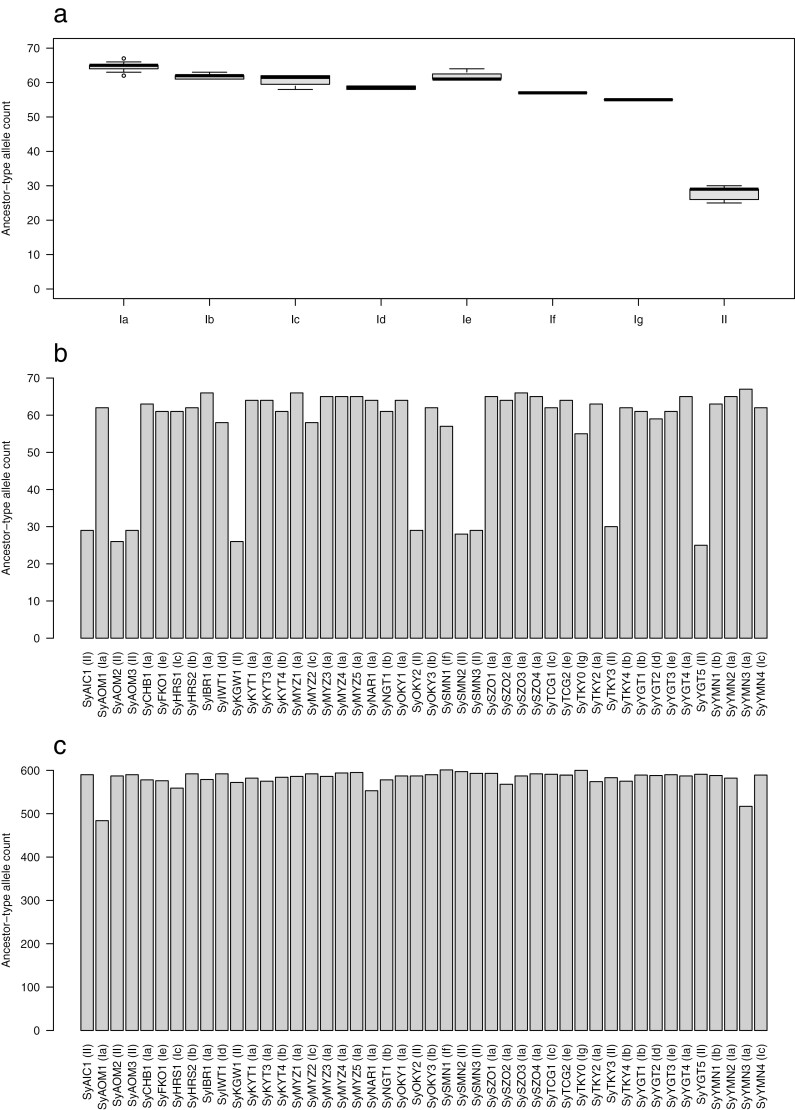
Ancestral alleles of ‘Somei-Yoshino’ clones. (a) Ancestral allele count in 71 common mutations for the eight groups of ‘Somei-Yoshino’ clones. (b) Ancestral allele count of ‘Somei-Yoshino’ clones in 71 common mutations at the sample level. (c) Ancestral allele count of ‘Somei-Yoshino’ clones in 613 unique mutations at the sample level.

To identify the group close to the potential ancestral type of ‘Somei-Yoshino’, genotypes of the 71 common variants in 46 samples were compared with those of 10 *Cerasus* lines belonging to at least six species, *C. campanulate* (Kanhi-zakura), *C. serrulate* (‘Azumanishiki’, ‘Gyoikou’, ‘Ichihara-toranowo’, and ‘Senrikou’), *C. spachiana* (‘Yaebeni-shidare’), *C. speciosa* (Ohshima-zakura), *C. jamasakura* (Yama-zakura), and *C.* × *nudiflora* (‘Eishu-zakura’) in addition to *Cerasus* sp. (‘Oshidori-fujizakura’). Since no variations were observed at the 71 sites of the 10 lines, the alleles possessed by the 10 lines were presumed to be the ancestor-type alleles of *Cerasus*. The mean values of the ancestor-type allele count in the eight groups were 64.6 (Ia), 61.7 (Ib), 60.8 (Ic), 58.5 (Id), 62.0 (Ie), 57.0 (If), 55.0 (Ig), and 27.9 (II) (Fig. 6a). At the sample level, the ancestor-type allele counts of the 71 common variants ranged from 25 (SyYGT5) to 67 (SyYMN3) (Fig. 6b) and those of the 613 unique variants were from 484 (SyAOM1) to 601 (SySMN1) (Fig. 6c).

## Discussion

A total of 684 somatic mutations were detected across 46 ‘Somei-Yoshino’ trees (Fig. 2, Supplementary Table S3). Because this mutation frequency was quite lower than that found among the flowering cherry accessions, the 46 trees were identified as clones of ‘Somei-Yoshino’ (Fig. 1, Supplementary Table S2). Of the 684 mutations, 71 were shared by multiple clones (Fig. 3, Supplementary Table S4). Since somatic mutations are seldom reversible, each of these 71 mutations would have a common origin and would be distributed across multiple lines via clonal propagation. Based on this analysis, the 46 ‘Somei-Yoshino’ clones were clustered into eight groups, Ia–Ig and II (Fig. 5). Interestingly, four trees collected from Ueno Park were classified into four different groups (Fig. 5b). Furthermore, 34 clones in addition to the four Ueno trees were included in the four groups (Fig. 5b). The ‘Somei-Yoshino’ clones tested in this study were collected from different locations across Japan, which might imply that ‘Somei-Yoshino’ trees in Japan might be mainly clustered into the four groups. Among the four trees planted in Ueno Park, SyTKY2 (tree ID #133), a member of group Ia, possessed the highest number of ancestor-type alleles among the four threes in Ueno Park (Fig. 6). This result suggested that SyTKY2 (tree ID #133) was the closest to the ancestral type. Although the actual origin of the clonally propagated ‘Somei-Yoshino’ was still unclear, we obtained a key set of somatic mutations to identify the origin. We hypothesize that the original tree could be a chimera composed of the somatic mutations found in the four groups. Since there are many candidates for the origin of ‘Somei-Yoshino’ in Japan^28^ as well as in Koishikawa Botanical Garden (Tokyo, Japan)^7^ and Kaiseizan Park (Fukushima, Japan),^9^ the origin could be discovered by finding the chimera.

The detected mutations consisted of 405 transitions and 279 transversions, with the transition/transversion ratio of 1.45. Among the different mutation types, the C/G to T/A transitions were the most prominent, the proportion of which (41.7%) was comparable with that of somatic mutations in popular^12^ and ethyl methanesulfonate (EMS)-induced artificial mutations in tomato.^29^ In addition, large-scale deletions (10–25 kb) were also found as somatic mutations, which have been found in not only chemical mutagenesis but also physical mutagenesis studies.^27,29^ Owing to these mutations, the functions of at least 13 and 23 genes might be severely and partially lost in the clones, respectively (Table 1, Supplementary Tables S3 and S4). Out of them, seven genes were reported as disease resistance-related genes, suggesting that these mutations could change disease resistance levels even in clonally propagated ‘Somei-Yoshino’. In several vegetatively propagated crops, mainly fruit trees, bud sports caused by somatic mutations were reported and used as new cultivars.^14^ For example, in grapes, a transposable element was reported as an inducer of a bud sport, in which the berry skin color was changed from black to white.^13^ However, few reports on phenotypic variations are available in ‘Somei-Yoshino’. Even though no deleterious mutations were found in genes involved in the flower opening mechanism,^5^ further investigation would be required to clarify whether the phenotypic variations are caused by genetic factors (somatic mutations) and/or environmental conditions.

The number of somatic mutations in the 46 trees was varied (Fig. 3, Supplementary Table S3). This variation in the number of somatic mutations was likely reflected by the number of unique variants rather than that of common variants, even though common variants were more frequent in group II than in group I (Fig. 3). The number of ancestor-type allele counts in the common variants might indicate the time of divergence from the ancestor, suggesting that the group II might be older than the other groups. On the other hand, the number of ancestor-type allele counts in the unique variants (Fig. 6c) might indicate the age of the clone after its propagation via cutting or grafting. It is believed that SyAOM1 is the oldest ‘Somei-Yoshino’ clone planted in 1888.^28^ Although the SyAOM2 tree is thought to be as old as the SyAOM1 trees, the ancestor-type allele counts of SyAOM2 was quite different from that of SyAOM1 (Fig. 6). SySZO1 tree is believed to have been obtained from Washington DC, USA, where ‘Somei-Yoshino’ trees from Japan were planted in 1912, but the number of ancestor-type allele counts in SySZO1 was similar to that in other clones (Fig. 6). In addition, although SyHRS2 is thought to have survived the atomic bomb attack in 1945 during World War II, the number of ancestor-type allele counts in this clone is not high (Fig. 6). Overall, the relationship between the ancestor-type allele counts and the age of ‘Somei-Yoshino’ was unclear.

In summary, we identified and characterized somatic mutations in ‘Somei-Yoshino’ clones collected from all over Japan. Since somatic mutations occur in different organs of an individual and are rarely reversed, leading to chimerism. Even if clonally propagated offspring basically inherit the mutations from the ancestors, their genotypes could be genetically divergent depending on the branches used for the propagation. Conversely, the somatic mutations could be used as tracers to find out the original tree. The somatic mutations found in this study could be key to identifying the origin of ‘Somei-Yoshino’, which has not been found to date. Furthermore, this somatic mutation-based tracing method could be used in agriculture to ensure the quality control of vegetatively propagated crops such as orange, apple, grape, strawberry, sweet potato, and tea, to protect the rights of breeders.

## Supplementary Material

dsae025_suppl_Supplementary_Tables_S1-S4

## Data Availability

The sequence reads are available from the DDBJ Sequence Read Archive (DRA) under the accession numbers DRR493485–DRR493529 of BioProject PRJDB16216.
